# A Systematic Review of Ecological Momentary Assessment of Diet: Implications and Perspectives for Nutritional Epidemiology

**DOI:** 10.3390/nu11112696

**Published:** 2019-11-07

**Authors:** Andrea Maugeri, Martina Barchitta

**Affiliations:** Department of Medical and Surgical Sciences and Advanced Technologies “GF Ingrassia”, University of Catania, Via S. Sofia 87, 95123 Catania, Italy; andreamaugeri88@gmail.com

**Keywords:** dietary assessment, epidemiology, foods, nutrients, obesity, eating behaviors

## Abstract

The ecological momentary assessment (EMA) of eating behaviors represents an innovative, detailed and valid approach to capture the complexity of food intake and to overcome limitations of traditional dietary assessment methods. Moreover, EMA studies might generate a large variety of data (e.g., dietary, behavioral, physical, sociopsychological, and contextual information), thereby enabling to examine concurrent exposures and events. Due to the increasing number of studies in this field of research, here we systematically reviewed EMA methods for the assessment of dietary intake in epidemiological studies, and discussed implications and perspectives for future research. Our study summarized several protocols and platforms that may be applied to assess diet in terms of eating frequency, choices, and habits. Nearly 38% of studies used an event-contingent strategy by asking participants to report foods and beverages consumed in real-time at each eating occasion. Instead, approximately 55% of studies used a signal-contingent prompting approach that notified the participants to record their dietary consumption. The remaining studies used a combination of event- and signal-contingent protocols to compare their accuracy or to improve the assessment of dietary data. Although both approaches might improve the accuracy and ecological validity of dietary assessment—also reducing the burden for participants—some limitations should nevertheless be considered. Despite these limitations, our systematic review pointed out that EMA can be applied in various fields of nutritional epidemiology, from the identification of determinants of dietary habits in healthy people to the management of patients with eating or metabolic disorders. However, more efforts should be encouraged to improve the validity and the reliability of EMA and to provide further technological innovations for public health research and interventions.

## 1. Introduction

In an article published in 2018 in the British Medical Journal (BMJ), Mozaffarian and colleagues have described how the history of modern nutritional science has shaped our understanding of diet and health [1]. To emphasize the importance of learning from historical events that have formed the basis of current research, the authors have even quoted Carl Sagan and Martin Luther King, Jr (i.e., “You have to know the past to understand the present” and “We are not makers of history. We are made by history”) [1]. However, while food and nutrition have been studied for centuries, modern nutritional science is unexpectedly young. Indeed, it is less than one century since the first vitamin was isolated in the 1920s, which has led researchers to focus on the role of micronutrients in deficiency diseases. More recently, some scientists began to indicate that examining the effects of foods and dietary patterns is more important for chronic diseases than a single nutrient approach [1].

In parallel with undeniably great strides in nutritional science, several methods for assessing dietary data—in terms of frequency of consumption, quality, and quantity—have been developed in the last decades. The common consensus is that biochemical and molecular biomarkers are the most objective surrogate estimates of dietary exposure since they are independent of memory and free of reporting biases [2]. However, while these markers are highly correlated with dietary intakes [3,4,5], they might be affected by individual metabolism, homeostatic regulation and genetic background [6,7,8]. Moreover, the information obtained through biochemical markers cannot be translated into specific dietary recommendations for patients [3,9]. Other objective methods include the duplicate diet approach and the collection of food records by trained researchers, but both are not suitable for large-scale epidemiological studies [2]. Among subjective dietary assessment methods, 24-h dietary recall and dietary records are open-ended surveys that collect a range of detailed information about food consumption (e.g., number of servings, portion size, cooking method, and brand name of commercial products) over a specific period [10]. The strength of 24-h dietary recalls is the relatively minimal burden imposed on respondents, but all information relies on their memory and on the skills of interviewer. In contrast, dietary records reduce reliance on respondents’ memory but require a high level of motivation of respondents [10,11]. The food frequency questionnaires (FFQs) are relatively simple, cost-effective, and time-efficient tools that have been used for assessing long-term dietary intake in large epidemiological studies since the 1990s. Yet, doubts on their accuracy were raised in the 2000s [1], a topic that is still debated among epidemiologists and nutritional professionals. Ioannidis—in an editorial published in 2018 in the Journal of the American Medical Association (JAMA)—has contributed to this debate and raised the need for reforming nutritional epidemiology by stating that current methodologies are inadequate and that randomized controlled trials do not support findings from observational studies [12]. The suggestion by Ioannidis is to adopt a transparent approach of nutrition-wide analyses of single nutrients, foods, and dietary patterns, which takes into account other exposures [12]. Indeed, dietary risks do not act alone on health but interact with other lifestyle factors, such as physical inactivity, alcohol abuse and smoking, which should be investigated simultaneously.

For these reasons, further efforts are required to develop innovative, thorough and valid approaches to capture the complexity of food intake and to overcome limitations of traditional dietary assessment methods. In our opinion, the ecological momentary assessment (EMA)—described for the first time by Shiffman and colleagues in 2008 [13]—has the potential to fill this gap by improving the validity and the reliability of dietary assessment. EMA was originally developed for the assessment of mood, emotions, and behaviors at the moment they occur in real life. The main features of EMA approaches are: (i) data collection occurs in the natural environment, (ii) the assessment refers to real-time information rather than retrospective surveys, (iii) the repeated sampling allows to define person’s current behaviors and experiences over a certain period [13]. Repeated sampling strategies include event-contingent or signal-contingent EMA, which can be used in combination with the support of an end-of-day survey [13]. The application of this real-time approach to dietary assessment differs from traditional retrospective methods, and both strategies (i.e., event- or signal-contingent EMA) might improve the accuracy and ecological validity by reducing reporting biases, recall interval and burden for participants. Moreover, EMA studies might generate a large variety of data, including dietary, behavioral, physical, sociopsychological, and contextual information, hereby enabling to examine concurrent exposures and events [14].

Due to the increasing number of studies in this field, we aimed to systematically review EMA methods for the assessment of dietary intake in epidemiological studies and to discuss implications and perspectives for future research. To reach this aim, we conducted a systematic review of epidemiological studies using EMA tool for assessing dietary intake and behaviors. Next, we summarized different EMA approaches and potential applications in the field of nutritional epidemiology.

## 2. Materials and Methods

### 2.1. Search Strategy

The authors performed a systematic literature search in the Medline, EMBASE, Web of Science, SCIELO, KCI Korean Journal, and Russian Science Citation Index databases from inception to June 2019. The literature search was limited to articles written in English. The following terms were used: (“ecological momentary assessment” OR “EMA”) AND (“diet” OR “food” OR “nutrient” OR “nutrition” OR “eating habits” OR “dietary habits” OR "dietary assessment" OR "nutrition assessment"). The Authors also searched the reference lists of selected articles to include all relevant studies. The preferred reporting items for systematic reviews and meta-analysis (PRISMA) guidelines were followed [15].

### 2.2. Selection Criteria and Data Extraction

The authors independently selected the retrieved studies if they were consistent with the following criteria: (i) observational or interventional epidemiological studies (ii) using EMA approaches (iii) to assess the characteristics of eating episodes (i.e., the number and type of each eating occasion) or the consumption of foods or beverages (expressed as dichotomous response, number of servings and/or portion size of specific foods, beverages, or food groups). By contrast, (i) systematic reviews, (ii) unpublished studies, (iii) and those that exclusively assessed eating disorders (e.g., binge eating or food craving) were excluded. From each study, the authors independently extracted the following information using a structured review template: first author’s last name, year of publication, study name, aims of research, platform, and device, monitoring periods and duration of EMA, prompting strategy (event- or signal-contingent or end-of-day report), and dietary data collected. For studies that applied signal-contingent EMA, the Authors also extracted information about prompting approach (random or fixed intervals), frequency, and period of validity of each prompt. If several articles were published on the same study, data were extracted from all articles and reported as a single study. Inconsistencies between the two Authors in the study selection and data extraction phases were resolved through discussion. The extracted data were synthesized narratively and tabularized with the intent of summarizing EMA protocols for assessing diet and to discuss their potential applications in nutritional epidemiology. The PRISMA checklist is reported as supplementary file.

## 3. Results and Discussion

### 3.1. Study Selection

**Figure 1:**
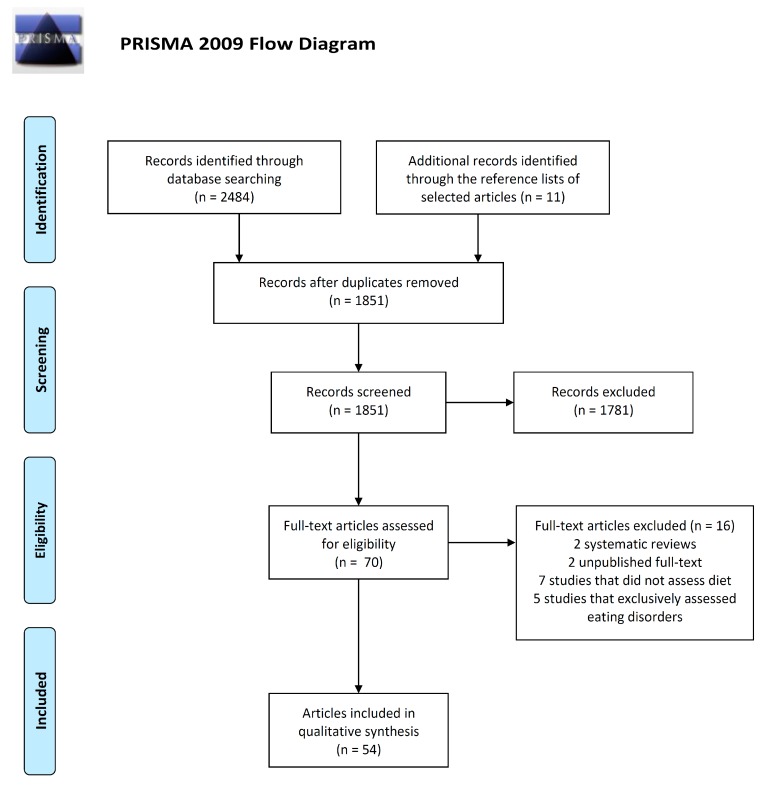
PRISMA flow diagram of study selection. Adapted from Moher D, Liberati A, Tetzlaff J, Altman DG, The PRISMA Group (2009). Preferred Reporting items for Systematic Reviews and Meta-Analyses: The PRISMA Statement. Plos Med 6(7):e1000097 [15].

Figure 1 shows the PRISMA flow diagram of study selection. After removing duplicates, a total of 1840 unique articles were retrieved from the databases, while 11 relevant articles were included from the reference lists of selected articles. Among these 1851 articles, 1781 were excluded after reading titles and/or abstracts. From those studies that underwent full-text screening, 16 were excluded according to selection criteria. Specifically, two systematic reviews, two unpublished full-text studies, seven studies that did not assess diet, and five studies that exclusively assessed eating disorders were excluded. Therefore, the systematic review included a total of 54 articles referring to 40 independent studies. In the next stages of the current systematic review, we extracted information from all 54 articles but reported data for independent studies (*n* = 40).

### 3.2. Sample Characteristics

The main characteristics of included studies are summarized in Table 1. In general, sample size ranged from 6 to 1450 participants and included mother-child pairs, pregnant women, children, and their families, adolescents or adults. While the majority of studies were conducted on healthy populations (*n* = 29), others recruited subjects with overweight or obesity (*n* = 6), anorexia nervosa (*n* = 2), type II diabetes (*n* = 2), or psychological disorders (*n* = 1).

### 3.3. Ecological Momentary Assessment Protocol

Approximately 63% of studies used electronic EMAs administered on smartphones (*n* = 25), 2 studies compared the reliability of a smartphone-based EMA approach with a paper-pencil recording, while the others used personal digital assistants (PDAs), web-based surveys or other devices. Almost all the studies monitored participants during one period of time (*n* = 37), while the remaining 3 studies included up to 6 waves of EMA. The duration of each monitoring period ranged from 3 days to 12 months, but about two-thirds of the studies (*n* = 24) monitored their participants from 7 to 14 days. With respect to EMA approaches, nearly 38% of studies (*n* = 15) used an event-contingent strategy by asking participants to report foods and beverages consumed in real-time at each eating occasion. Instead, approximately 55% of studies (*n* = 22) used a signal-contingent prompting approach that—through researcher-initiated signaled prompts—notified the participants to record their dietary consumption.

Contrary to the event-contingent approach, signal-contingent EMA did not always allow for the real-time assessment of diet but rather included questions about the consumption of foods or beverages occurred within a recent interval of time. The remaining 3 studies used a combination of event- and signal-contingent protocols to compare their accuracy or to improve the assessment of dietary data. Interestingly, 4 studies also administered in the evening an end-of-day survey to report on any food consumed that had not been previously recorded.

### 3.4. Prompting Strategies of Signal-Contingent EMA

Among studies that applied a signal-contingent protocol, 11 used a fixed interval schedule based on mealtimes and/or daily routine of each participant, whereas 14 used a random schedule within fixed or semifixed time intervals. The frequency of sampling of signal-contingent EMAs was determined by the researcher and ranged from 1 to 14 prompts per day. Notably, the study with the longest monitoring period prompted their participants 1 time per day up to one year, while the prompt frequency was higher in studies with the shortest duration. Several studies had different prompting frequencies during the week, with more prompts during weekend days than weekdays. The type of notification of each prompt depended on the device used for EMA: in general, smartphone-based EMAs notified their participants by text messages or mobile notifications, while the others used also emails or beeps from palm devices. The validity of each prompt ranged from 6 to 90 minutes, while 1 study did not give any reminder.

### 3.5. Dietary Data Collected

Overall, 8 event-contingent EMA studies asked their participants to define the type of each eating episode (e.g., meal, snack or binge eating). Among these, 4 studies also asked to define the consumption of specific foods or food categories from a predefined list. Instead, another study exclusively recorded the consumption of predefined food types or food groups. The other 5 studies that used an event-contingent protocol asked their participants to capture images or videos of each eating episode, sometimes followed by a brief text/voice description (*n* = 3). The study by Hingle and colleagues was one of a kind, collecting consumption of foods and beverages using hashtags on Twitter [32]. With respect to the signal-contingent approach, the majority of studies recorded the consumption and/or the number of servings of predefined foods or food categories (*n* = 15). The remaining studies asked their participants to define the type of each eating episode (*n* = 4), to report frequency of eating and snacking (*n* = 1), or to capture food images (*n* = 2). Notably, the latter studies used food images to assess nutrient or total energy intake.

### 3.6. Remarkable Findings and Implications in Nutritional Epidemiology

**Figure 2:**
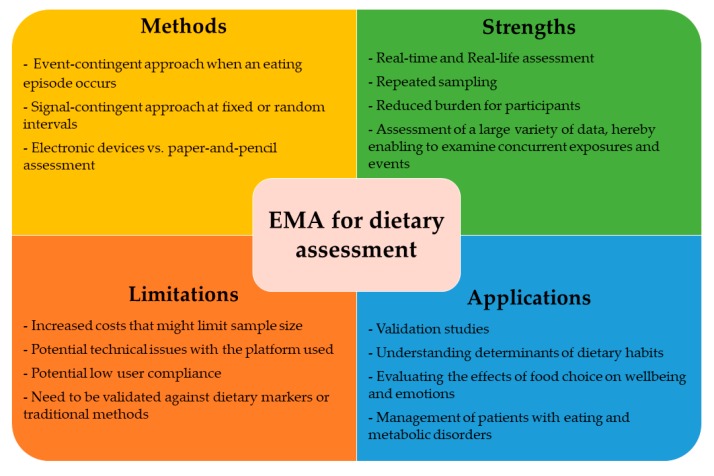
Characteristics of ecological momentary assessment of diet and potential applications in nutritional epidemiology.

To the best of our knowledge, our study provides the most accurate and complete systematic review of EMA protocols used for the assessment of dietary intake in epidemiological research. Indeed, previous reviews have focused only on mobile EMA or specific age groups (e.g., children and adolescents) [65,66]. At such time as current methodologies are extremely debated, our impression is that applying EMA protocols may improve the validity and reliability of dietary assessment. Potential applications of EMA are outlined in Figure 2 and described in the following paragraphs.

#### 3.6.1. Validation Studies

The first thing we noticed was that few studies validated EMA protocols against current dietary assessment methods or nutritional markers. Among these, Chmurzynska and colleagues compared the feasibility of a signal-contingent EMA protocol for measuring the frequency of consumption of high-fat foods with standard retrospective methods, such as the Block Screening Questionnaire for Fat Intake [20]. Interestingly, they reported that using EMA improved the assessment of dietary data, especially in overweight or obese individuals [20]. Next, Bucher Della Torre and colleagues developed the electronic carnet alimentaire (e-CA)—“food record” in French—which included approximately 900 foods and beverages classified in 14 categories [19]. They evaluated its usability and acceptability, accuracy, and reliability in a multistage process. Specifically, the comparisons of e-CA with 24-h recalls and food records were reported acceptable but were also highlighted limitations in estimating portion sizes [19]. Other studies that tested the validity of their EMA protocols were those that asked their participants to capture food images or videos. Martin and colleagues were the first to demonstrate the validity of a signal-contingent EMA approach for estimating energy intake through food images, against doubly labeled water method [33]. Similarly, Boushey and colleagues validated their image-based event-contingent EMA of total energy intake among community-dwelling adults [18]. An image-based record may impose a relatively minimal burden on participants if compared with traditional dietary assessment methods, especially in those with peculiar conditions. For instance, a real-time assessment of dietary intake in pregnant women might help us confirm current evidence about the main determinants of diet during pregnancy [67], and its effects on pregnancy outcomes [68,69]. With the goal of supporting nutrition professionals, Ashman and colleagues developed an image-based, event-contingent assessment of dietary intake among pregnant women and assessed its relative validity against three 24-h recalls [16]. The proposed tool showed an acceptable relative validity for establishing dietary intake, accompanied by good usability and acceptability [16]. However, despite these strides, we are still far from applying EMA protocols in nutritional epidemiology continuously. Indeed, other studies concluded that these innovative methods still need further adjustments [37].

#### 3.6.2. Family Environment and Dietary Habits

As mentioned above, women have a crucial role in food choice by providing meals for their families and making up the majority of the workforce in food-related jobs, health care and education [70]. Thus, two independent studies—the Toddler Overweight Prevention Study (TOPS) and the Mothers and Their Children’s Health (MATCH) study—aimed to identify factors in the home environment associated with child diet and physical activity [38,60].

The TOPS recruited 277 mother–child pairs and applied a random signal-contingent assessment of child consumption of meals, snacks or drinks [60]. In 2018, the authors identified several factors in the home environment that were associated with dietary behaviors among children [59]. The MATCH was a longitudinal, observational, dyadic, case-crossover study, which monitored 200 mother-child pairs for 6 semi-annual assessment waves across three years [38]. Using a random signal-contingent assessment of food consumed—(i.e., fries, sweets, fast foods, fruit or vegetables, energy drinks), the researchers first compared the effects of hypercaloric foods versus fruits and vegetables on maternal affective states, demonstrating that the consumption of high-fat and high-sugar foods increased stress levels, especially in overweight and obese women [39]. Next, they evaluated the concordance of children’s dietary reports through EMA and 24-h recall in 144 children, achieving good results but improvable [41].

This tool was also applied to examine mothers’ and children’s dietary intake during physical and sedentary activities [40]. Experienced gained by a group of researchers from the MATCH study has allowed them to design the Maternal and Developmental Risks from Environmental and Social Stressors (MADRES) study [34]. Their proposal is to apply an innovative study protocol—which integrates EMA, personal exposure monitoring, geo-localization, and accelerometry—to examine the daily effects of environmental and social factors on obesity-related behaviors in pregnant women [34]. In our opinion, one of the best examples of dietary assessment in the family environment is represented by the Family Matters study [71]. This study aims to identify individual, dyadic and familial factors associated with childhood obesity through a two-phased incremental, mixed-methods, and longitudinal approach [71]. The study protocol used an event-contingent EMA to assess the consumption of homemade, pre-prepared and fast foods in 150 children aged 5 to 7 years and their families [71]. Several articles have been published by the Family Matters researchers, which described the main familial feeding practices that might affect parents’ and children’s health [72,73,74,75].

#### 3.6.3. Determinants of Unhealthy Eating among Young People

In 2013, two independent research groups were the first to assess dietary habits in young people through an EMA approach. In particular, Spook and colleagues examined the feasibility, usability, and ecological validity of a mobile EMA application to assess physical activity and dietary intake among Dutch vocational education students [56]. Although the proposed tool offered the opportunity to assess complex health behaviors in real-time, it exhibited several limitations that needed to be solved [56]. Similarly, Grenard and colleagues developed an event-contingent assessment of eating episodes administered through a personal digital assistant [30]. Interestingly, they demonstrated that the consumption of sweetened drinks and snacks was associated with several social and intrapersonal cues, such as being at school or with friends, watching TV, feeling lonely or bored, craving a drink or snack, and being exposed to food cues [30].

Food craving—an intense desire to eat a specific food—is certainly one of the main triggers of an exaggerated intake of energy-dense foods, such as snacks and dessert foods. Plausible explanations include ingredients of certain foods (e.g., phenylethylamine in chocolate), low serotonin levels affecting the centers for appetite, or the release of endorphins as a result of a state of stress [76]. To address this topic, Berkman and colleagues compared compliance—in terms of response rate and response latency—between electronic and paper-and-pencil methods to assess the number of servings of a selected energy-dense food (e.g., snack or dessert food) [17]. Interestingly, the compliance was higher for the electronic EMA than the paper-and-pencil assessment, with better response rate, response latency and lesser influence by BMI [17]. More recently, Richard and colleagues developed a signal-contingent EMA to characterize snacking and food craving among female students [45]. They first demonstrated that higher craving intensity and more snack-related thoughts were associated with more consumption of snacks and that chocolate containing foods were the most often reported snacks [45]. Next, they showed that a more positive implicit evaluation of chocolate was associated with higher likelihood of consuming it in states of hunger or craving [46].

Food craving may also dangerously lead to uncontrolled overeating, especially in overweight and obese people. Despite this evidence, contextual factors related to maladaptive eating behavior in youth still remain poorly clarified. To examine internal and external cues related to perceptions of overeating and loss of control Goldschmidt and colleagues integrated different EMA approaches among 40 overweight or obese children [29]. Specifically, they used an event-contingent assessment of each eating episode, accompanied by signal-contingent prompts and end-of-day surveys to report on any eating episode that had not been previously recorded [29]. Their findings pointed out that eating-related factors (e.g., perceived palatability of food being consumed) were strongly associated with loss of control severity, while contextual factors (e.g., location or eating with others) were associated with overeating severity [29]. Interventions aimed to treat maladaptive eating in children and adolescents, especially in those with overweight or obesity, may benefit from incorporating palatable foods, and enhancing awareness of social-contextual effects on eating. With this in mind, the interventional study by Forman and colleagues applied a signal-contingent EMA to test the effects of computerized inhibitory control (ICT) and mindful decision-making (MDT) training in reducing food craving [24]. Accordingly, the authors randomized 119 habitual salty snack eaters to one of four training conditions: ICT, MDT, and ICT and MDT, or psychoeducation. Their findings demonstrated that benefits from the MDT were similar across different levels of emotional eating, while those from the ICT were significant only at lower levels [24].

From a broader perspective, some studies applied EMA strategies to evaluate the main determinants of unhealthy behaviors among students. In this context, to the best of our knowledge, one of the largest studies is the Social impact of Physical Activity and Nutrition in College (SPARC) study, which recruited 1450 first-year college students over an academic year [55]. The main aim of the SPARC study was to determine mechanisms by which friendship networks impact eating, physical activity, and weight. Each of four waves consisted of four quasi-randomly selected days of a signal-contingent assessment of food consumption (i.e., eight food categories and eight types of beverage) [55]. They first validated the devil SPARC mobile EMA application for assessing eating behaviors and sedentary activity at the day level [54]. Next, they demonstrated that both negative and positive emotions were significantly associated with food choices: specifically, at the within-person level, negative emotions were associated with the consumption of meat/proteins, while positive emotions were associated with the consumption of sweets [53]. Their findings might be helpful to guide further prospective studies and to propose novel strategies that encourage healthy food choices in youth.

#### 3.6.4. Factors that Prompt to Snacking

Meal frequency and snacking vary dramatically across cultures, change with time, and may profoundly affect metabolic and cardiovascular health [77,78]. A reasonable effect of between-meal snacking consisted of increased total energy intake [77]. To develop novel methods for assessing energy from snacks, Wouters and colleagues compared a smartphone-based signal-contingent tool with an event-contingent paper and pencil diary among 46 students [62]. Although they reported comparable results in momentary energy intake from snacks between the two methods, a significant difference was evident for energy intake from total daily snack consumption [62]. These findings indicate that strengths and limitations of each EMA approach should be considered when designing a study protocol, according to research purpose and sampling procedure.

Other studies used EMA for understanding the main determinants of snacking and its relationship with health and diseases. Indeed, every day, we are faced with external stimuli (e.g., food items in shop displays, advertisements for food, or seeing other people eat) that prompt us to eat. In 2015, Schüz and colleagues provided the first attempt to describe environmental and affective factors that were associated with eating and drinking using an EMA approach [48]. For instance, they showed that snacking was associated with negative affect, having food available, and observing others eat [49]. Particularly, these findings supported the idea of comfort eating, confirming that effects and emotions may shape eating behavior. In a further study, Schüz and colleagues aimed to examine whether individual differences in cue effects on snacking can be explained by the Power of Food Scale (PFS), which was designed to measures the awareness of food availability, reactions to thinking about food, and reactions to tasting food [47]. Interestingly, people with higher PFS scores were more likely to snack when experiencing negative affect, high arousal, engaging in activities, and being alone. This puts them at higher risk for unhealthy and obesogenic eating behavior [47].

The cognitive processes responsible for effortful behavioral regulation are known as executive functions. The Snacking, Physical activity, Self-regulation, and Heart rate Over Time (SNAPSHOT) study applied an EMA approach to examine the relationship between snacking, energy expenditure, and executive function [52]. Results from this study—published by Powell and colleagues in 2017—confirmed the current opinion that inhibitory control played a key role in prompting snacking, pointing out it as an important target for interventions [51]. More recently, Ghosh Roy and colleagues examined associations of contextual factors with within-person variations in snack food and sweetened beverage intake among African American women [25]. They asked their participants to wear a global positioning system (GPS) logger and to complete a signal-contingent EMA of behaviors and environmental, social, and other contextual factors [25]. Notably, perception of close proximity to fast-food restaurants and convenience stores was associated with increased odds of snacking [25]. This was partially in line with results reported by Zenk and colleague in 2014 [64]. Moreover, women who engaged in activities while eating (e.g. watching television and talking) were more likely to consume snacks [64]. Overall, these findings raise the need for public health strategies addressing fast-food restaurants and convenience store accessibility, food offerings and marketing to help reduce snack food intake.

#### 3.6.5. Effects of Food Choices on Wellbeing and Emotions

While several behaviors, such as physical activity and sleeping, have been shown to have stress-reducing and beneficial effects on wellbeing and health [79,80,81], the rewarding effect of eating has rarely been investigated. In 2017, an EMA study by Strahler and Nater demonstrated that the consumption of juice, coffee, and alcohol improved momentary wellbeing while snacking reduced fatigue levels [57]. By contrast, the consumption of high-fat foods resulted in impaired wellbeing, contrasting with the belief that high-caloric foods taste better, make us happy, and alleviate a negative mood [57]. In the same year, Wahl and colleagues added to this topic showing that healthy food choices—such as eating fruits and vegetables—were associated with eating happiness [61].

Understanding the relationship between behaviors and emotions might be of particular interest in people with physical or mental health disorders. For instance, researchers of the Mobile Community Health Assistance for Tenants (m.chat) project used EMA to investigate the association of daily emotional states with physical activity, diet, social interaction, medication compliance, and tobacco smoking, among people with a history of chronic homelessness who participated in a health coaching program [36]. Healthy behaviors, such as physical activity and consumption of fruits and vegetables, generally enhanced positive affect and restrained negative affect. Specifically, a greater number of servings of fruits and vegetables was associated with higher scores for valence and arousal [36].

#### 3.6.6. Management of Patients with Eating Disorders, Obesity or Diabetes

The first studies using EMA approaches in disease states were those related to anorexia nervosa, which increased our knowledge of different emotional, behavioral, and environmental features that affect disturbances in eating behavior. In 2014, Goldschmidt and colleagues asked women with anorexia nervosa to complete an event-contingent EMA of daily eating- and mood-related patterns [26]. Their findings supported the presence of discrete types of eating episodes that were associated with negative affect, stress, and behavioral features of eating disorders among patients with anorexia nervosa [26]. In the same population, Haynos and colleagues evaluated whether restrictive eating served an emotional avoidance function among individuals with anorexia nervosa [31]. However, they concluded that both negative and positive moods were not significantly different between days characterized by high restriction and those characterized by low or no restriction [31]. Researchers from the same group applied a similar approach to examine contextual factors associated with eating in the absence of hunger among adults with obesity [27]. They improved their EMA approach by adding a signal-contingent assessment and an end-of-day survey to report on eating episodes that had not been previously recorded [27]. Interestingly, at the within-person level, eating in the absence of hunger was associated with greater overeating.

Yet, at the between-person level, people who commonly ate in the absence of hunger reported less overeating. Moreover, eating in the evening was associated with several contextual factors, such as overeating, alcoholic drinking, eating alone, eating because others are eating, and eating while watching television [27]. Next, the researchers aimed to evaluate the association between binge-eating and five features of binge-eating episodes that were described by the Diagnostic and Statistical Manual of Mental Disorders [28]. Among them, binge-eating seemed associated with lower pre-episode hunger, higher post-episode fullness, eating alone, and feeling disgusted, depressed, and guilty after eating [28]. Beyond eating disorders, contextual factors may also affect every day eating behavior. Thus, Elliston and colleagues used an event-contingent EMA to examine situational cues and momentary food environment that influenced eating decisions in adults with overweight and obesity [22]. Their findings indicated that both internal (i.e., affect) and external (i.e., food availability, social context, observing others eating) cues increased the odds of eating. Particularly, the perception of close proximity to fast-food restaurants or supermarkets influenced food choices [22].

This raises the need for an integrated approach to understanding those factors that affect eating behavior, which may help us in improving preventive strategies against the obesity epidemic. Further studies used EMA to prepare obese patients for bariatric surgery [42] or to monitor their postoperative eating and activity behaviors [58]. Mundi and colleagues used a smartphone-based signal-contingent EMA to assess recommended dietary behaviors and to support healthy food choices, among obese patients scheduled for primary bariatric surgery [42]. Although more efforts are necessary to confirm its efficacy, this tool was satisfactory and tended to promote behavioral changes and increased weight loss among patients [42]. Since successful weight loss depends on postoperative compliance with recommended behaviors, Thomas and colleagues used an event-contingent EMA to assess eating habits of patients who underwent bariatric surgery [58]. Participants demonstrated good compliance with the EMA, which in turn proved to be an useful tool to identify behavioral targets for postoperative monitoring and interventions [58].

With respect to diabetes, a pilot study by Miller and colleagues aimed to monitor the adoption of a lower glycemic index diet among six patients with type II diabetes for six weeks [35]. Using a signal-contingent EMA of low glycemic index foods, the authors reported three patients failed in achieving their goal [35]. Similarly, Waki and colleagues designed a randomized study to assess the feasibility of a remote health-data monitoring system to modify patient behaviors and to improve clinical outcomes [63]. This system included an event-contingent EMA of each eating occasion by taking food images. After three months of follow-up, HbA1c and fasting glucose levels decreased in the intervention group, while increased in the non-intervention group [63]. Therefore, this tool seemed to support diabetic patients in improving their clinical outcomes. However, larger randomized controlled trial should be encouraged to confirm its feasibility and efficacy in a broader context.

## 4. Conclusions

Our systematic review summarizes current EMA methods used for the assessment of diet in nutritional epidemiology, and their potential applications for understanding the relationships between eating behaviors, health, and disease. When deciding to design an EMA study, several protocols and platforms may be applied to assess diet in terms of eating frequency, choices, and habits. We found that the majority of studies used smartphone-based EMA, even if other devices may be applied. The use of smartphones and other electronic devices definitively enhances the applicability and validity of EMA in nutritional epidemiology. However, it also poses some challenges for scientists and their funds. Indeed, EMAs administered through electronic devices are more expensive than other paper-and-pencil surveys, especially for those studies that provide participants with loaned devices. Whilst this ensures consistency in the administration of EMAs, it also may limit overall sample size. An alternative would be to use open source software, which allows tailoring EMA protocols to specific research topics. Moreover, several EMA protocols and prompting strategies might be applied, including event-contingent and signal-contingent approaches, which can be occasionally supported by an end-of-day survey. Although both might improve the accuracy and ecological validity of dietary assessment—also reducing the burden for participants—some limitations should nevertheless be considered. For instance, technical issues with the platform used or low user compliance might impair the assessment of dietary intake. The majority of studies included in the present systematic review did not report user response rate and compliance. However, it has been reported that compliance was higher in electronic groups than in people who received paper-and-pencil survey [17]. By contrast, electronic EMA might be less acceptable to older people who are less comfortable with smartphone-based technologies or those with low electronic literacy. Moreover, it has been also demonstrated that daily compliance declined during the monitoring period [17]. Finally, for studies that used event-contingent EMA, it was difficult to determine the compliance rate, since people were asked to report foods and beverages consumed at each eating occasion, without a predefined number of diary prompts that participants were required to complete. Therefore, our suggestion is to administer an end-of-study survey to collect information about acceptability and usability and to provide this information for allowing readers to critically assess scientific and technical soundness. In our opinion, a proactive approach should be applied to maintain regular contact with participants. For instance, participants could be provided with small gifts and/or reimbursements, which serve both as an expression of gratitude for continued participation and to maintain subject contact.

Despite these limitations, our systematic review points out that EMA can be applied in various fields of nutritional epidemiology, from the identification of determinants of dietary habits in healthy people to the management of patients with eating or metabolic disorders. However, more efforts should be encouraged to improve the validity and the reliability of EMA, overcoming limitations of traditional dietary assessment methods, and to provide further technological innovations for public health research and interventions. 

## Figures and Tables

**Table 1:** Characteristics of studies included in the systematic review.

Study Name or First Author and Year of Publication	Study Population	Aims of Research	Platform and Device	Duration of EMA	EMA Procedures	Dietary Data Collected	References
Ashman 2017	25 pregnant women aged 20 to 50 years	To assess the relative validity of image-based dietary records for assessment of intake among pregnant women	Smartphone	3 days	Event-contingent assessment whenever an eating occasion occurred	Phone images of all eating and drinking occasions, with a brief text/voice description	[16]
Berkman 2014	44 young adults aged 18 to 30 years	To compare two EMA methods to examine food craving, and to assess their sensitivity to individual difference variables such as body mass index	Paper-and-pencil vs. text messaging by smartphone	14 days	Signal-contingent assessment at mealtime-based intervals	Number of servings of a selected energy-dense food (e.g., snack or dessert food)	[17]
Boushey 2017	45 adults aged 21 to 65 years	To test the accuracy of a mobile app by comparing reported energy intake to total energy expenditure using the doubly labeled water method	Smartphone	7.5 days	Event-contingent assessment whenever an eating occasion occurred	Phone images of all eating occasions	[18]
Bucher Della Torre 2017	Three study phases including 10, 18 and 22 adults, respectively	To develop and evaluate an electronic mobile-based food record for a research setting	Web-based survey	4-5 days	Event-contingent assessment whenever an eating occasion occurred	Number of servings and portion sizes of foods and beverages chosen from 900 options	[19]
Chmurzynska 2018	62 adults aged 20 to 40 years	To evaluate the feasibility of an application for measuring the frequency of consumption of high-fat foods	Smartphone	7 days	Signal-contingent assessment at fixed intervals	Consumption of high-fat foods	[20]
Comulada 2018	42 women, aged 20 to 43 years, having a child under 18 years of age living at home	To examine the adherence to the use of a mobile app designed to help mothers self-monitor lifestyle behaviors and stress	Android smartphone	6 months	Signal-contingent assessment at random intervals	Type of eating episodes (i.e., meal or snack)	[21]
Elliston 2016	51 overweight or obese adults, aged 19 to 73 years	To examine the influence of both cues and the momentary food environment on real-time eating decisions in adults with overweight and obesity	Android smartphone	14 days	Event-contingent assessment whenever an eating occasion occurred	Reporting on eating episode (meal or snack) and food category (i.e., fruit and vegetables, starchy foods, fish, chips, meat, meat products, poultry, cheese, sweets or chocolates, ice cream, crisps/savory snacks, cakes/scones/pastry, biscuits) of foods consumed	[22]
Family Matters study	150 children aged 5 to 7 years and their families	To identify novel risk and protective factors for childhood obesity in the home environments of racially/ethnically diverse and primarily low-income children	iPad mini	8 days	Event-contingent assessment whenever an eating occasion occurred	Consumption of homemade, pre-prepared or fast foods	[23,24,25,26,27]
					End-of-day survey	Reporting on any food consumed that had not been previously recorded	
Fly-in Fly-out Lifestyle EMA study	64 fly-in, fly-out workers (mean age = 40.4 years) and 42 partners (mean age = 38.6 years)	To examine health behavior patterns of Fly-in, fly-out workers and their partners during on-shift and off-shift time frames	Web-based survey	14 days	Signal-contingent assessment at fixed intervals	Number of alcoholic drinks per day	[23]
Forman 2016	119 undergraduate students aged 18 to 47 years	To test the independent and combinatory effects of two mindful decision-making training and inhibitory control training on consumption of hedonic eating	Smartphone	14 days	Signal-contingent assessment at fixed intervals	Number of snack servings consumed	[24]
Ghosh Roy 2019	101 women aged 25 to 65 years	To explore within-person associations between contextual factors and intake of energy-dense snack foods or sweetened beverages	Smartphone	7 days	Signal-contingent assessment at random intervals	Consumption of snacks (i.e., fries, salty snacks, cookies or sweetened baked good, ice cream) and sweetened beverage	[25]
Goldschmidt 2014	118 women with anorexia nervosa, aged 18 to 58 years	To examine the emotional and behavioral context in which several classes of eating episodes occur. A secondary aim was to examine the extent to which anorexia nervosa diagnostic subtypes differed with respect to self-reported frequencies of different classes of eating episodes	Palm device	14 days	Event-contingent assessment whenever an eating occasion occurred	Reporting on eating episode (meal, snack or binge eating)	[26]
Goldschmidt 2017	50 obese adults, aged 18 to 65 years	To examine associations between the Diagnostic and Statistical Manual for Psychiatric Disorders indicators and binge versus non-binge episodes	Smartphone	14 days	Event-contingent assessment whenever an eating occasion occurred	Reporting on eating type episode (meal, snack or binge eating)	[27,28]
					Signal-contingent assessment at random intervals	Reporting on any recent eating episode that had not been previously recorded	
					End-of-day survey	Reporting on any recent eating episode that had not been previously recorded	
Goldschmidt 2018	40 overweight or obese children aged 8 to 14 years	To elucidate immediate internal and external cues related to perceptions of overeating and loss of control overeating	Smartphone	14 days	Event-contingent assessment whenever an eating occasion occurred	Reporting on eating episode (meal, snack or binge eating)	[29]
					Signal-contingent assessment at random intervals	Reporting on any recent eating episode that had not been previously recorded	
					End-of-day survey	Reporting on any recent eating episode that had not been previously recorded	
Grenard 2013	158 students aged 14 to 17 years	To identify physical, social, and intrapersonal cues that were associated with the consumption of sweetened beverages, sweets, and salty snacks	Palm device	7 days	Event-contingent assessment whenever an eating occasion occurred	Reporting on eating episode (meal, snack) and food categories (i.e., lists of drinks, snacks, fruit/vegetables, carbohydrates, protein, and meat)	[30]
Haynos 2015	118 women who met criteria for anorexia nervosa	To investigate whether restrictive eating serves an avoidance function among individuals with anorexia nervosa	Palm device	14 days	Event-contingent assessment whenever an eating occasion occurred	Reporting on eating episode (meal, snack or binge eating)	[31]
Hingle 2013	50 adults	To test the feasibility and acceptability of Twitter to capture young adults’ dietary behavior and reasons for eating	Web-based survey	3 days	Event-contingent assessment whenever an eating occasion occurred	Recording of foods and beverages consumed using Twitter application	[32]
Martin 2012	50 adults aged 18 to 65 years	To test the reliability and validity of the Remote Food Photography Method to estimate energy and nutrient intake	Smartphone	6 days	Signal-contingent assessment at mealtime-based intervals	Food images for assessing energy and nutrient intake	[33]
Maternal and Developmental Risks from Environmental and Social Stressors (MADRES) study	65 pregnant women aged 18 years or older	To examine the daily effects of environmental and social stressors on maternal pre- and post-partum obesity-related biobehavioral responses	Android smartphone	3 waves of 4 days during the 1st and 3rd trimester, and at 4–6months postpartum	Signal-contingent assessment at random intervals	Consumption of foods (i.e., fries, sweets, fast foods, fruit or vegetables, energy drinks)	[34]
Miller 2016	6 adults with type II diabetes mellitus, aged 58 to 62 years	To ascertain goal pursuit toward the adoption of a lower glycemic index diet among adults with type II diabetes mellitus	Wrist-worn electronic diary	6 weeks	Signal-contingent assessment at random intervals	Number of servings of low glycemic index foods	[35]
Mobile Community Health Assistance for Tenants (m.chat) project	155 adults (mean age = 52 years) who reported prescribed medication for psychological or emotional problems, or experienced hallucinations, or received a pension for a psychiatric disability, or reported at least moderate levels of depression	To assess the feasibility of technology assisted health coaching intervention designed to improve health indicators among permanent supporting housing individuals	Smartphone	Up to 12 months	Signal-contingent assessment at fixed intervals	Number of servings of fruits, vegetables, sugar-sweetened beverages, desserts and other sweets the previous day	[36]
Most 2018	23 obese pregnant women, aged 18 to 40 years	To evaluate the accuracy of an electronic reporting method to measure daily energy intake	Smartphone	6 days	Signal-contingent assessment at mealtime-based intervals	Food images for assessing energy intake	[37]
Mothers And Their Children’s Health (MATCH) study	200 mothers with their 8–12-year-old children	To examine within-day associations of maternal stress with children’s physical activity and dietary intake, and how these effects contribute to children’s obesity risk	Android smartphone	6 semi-annual waves across 3 years. Each wave consists of 7-day EMA assessment	Signal-contingent assessment at random intervals	Consumption of foods (i.e., fries, sweets, fast foods, fruit or vegetables, energy drinks)	[38,39,40,41]
Mundi 2015	30 adults who underwent evaluation for primary laparoscopic bariatric surgery (mean age = 41.3 years)	To assess feasibility of using smartphone app with EMA functionality to prepare patients for bariatric surgery	Android or iPhone smartphones	Up to 15 weeks	Signal-contingent assessment at random intervals	Frequency of eating and snacking. Frequency of use of calorie-containing beverages. Meal planning. Frequency of foods not prepared at home	[42]
Reader 2018	57 undergraduate students, aged 18 to 22 years	To estimate the relative efficacy in reappraising high-calorie foods with reappraisal of low-calorie food items, and to relate reappraisal efficacy measures to real-world consumptive behavior	Smartphone	7 days	Signal-contingent assessment at random intervals	Consumption and the amount of craved foods	[43]
Reichenberger 2018b	59 adolescents or adults, aged 14 to 65 years	To evaluate the effects of stress, negative and positive emotions on taste- and hunger-based eating	Smartphone	10 days	Signal-contingent assessment at random intervals	Reporting on eating episode (meal, snack or binge eating)	[44]
Richard 2017	66 female university students aged 18 to 30 years	To characterize food craving in real life	Smartphone	7 days	Signal-contingent assessment at fixed intervals	Number of consumed snacks	[45,46]
Schüz 2015	53 adults aged 18 to 60 years	To examine every day snacking using real-time assessment, and to test if individual differences in cue effects on snacking can be explained by the Power of Food scale	Smartphone	10 days	Event-contingent assessment whenever an eating occasion occurred	Reporting on eating episode (meal or snack)	[47,48]
					End-of-day survey	Reporting on any eating episode that had not been previously recorded	
Schüz 2017	112 adults aged 18 to 73 years	To explore whether there are BMI-related differences in individual snacking behavior following social cues in a real-world setting	Smartphone	14 days	Event-contingent assessment whenever an eating occasion occurred	Reporting on eating episode (meal or snack) and the type of snack (i.e., fruit/nuts, vegetables, dairy, or higher-energy snacks)	[49]
Seto 2016	12 students	To address the multitude of factors that affect obesity, including unhealthy individual behaviors and environmental characteristics	Android smartphone	6 days	Event-contingent assessment whenever an eating occasion occurred	Voice-annotated video to assess food consumed and portion size	[50]
SNAcking, Physical activity, Self-regulation, and Heart-rate Over Time (SNAPSHOT) project	64 adults aged 18 to 70 years	To track inhibitory control and snacking behavior in real time to test a series of novel hypotheses regarding the relationship between executive function and dietary control	Wrist-worn electronic diary	7 days	Signal-contingent assessment at fixed intervals	Consumption and number of snacks, fruits and vegetables	[51,52]
Social impact of Physical Activity and nutRition in College (SPARC) study	1450 first-year college students	To determine mechanisms by which friendship networks impact eating, physical activity and weight	Android or iPhone smartphones	4 waves during the 1-year period. Each wave consists of 4 quasi-randomly selected days throughout a 7-day period	Signal-contingent assessment at random intervals	Consumption of foods (8 food categories) and drinks (8 types of beverages)	[53,54,55]
Spook 2013	30 students aged 16 to 21 years	To examine the feasibility, usability and ecological validity of a mobile app for assessing determinants of diet and physical activity	Smartphone	7 days	Signal-contingent assessment at fixed intervals	Frequency, type and amount of foods consumed (i.e., fruits and vegetables, snacks and sodas)	[56]
Strahler 2017	77 adults (mean age =23.9 years)	To measure intake of food and drink close to real-time, and to provide an ecologically valid approach to examine their predictive role in the context of wellbeing	iPod Touch	4 days	Signal-contingent assessment at fixed intervals	Reporting on eating type (i.e., main dish, snack, sweet, other) and its main component (i.e., protein, carbohydrate, fat, mixed), and drink consumption (i.e., water or unsweetened tea, sweetened drinks, juice, caffeinated drinks, or alcoholic beverages)	[57]
Thomas 2011	21 patients who underwent laparoscopic adjustable gastric banding or Roux-en-Y gastric bypass	To assess bariatric surgery patients’ eating and activity behaviors in real-time in the natural environment	Palm device	6 days	Event-contingent assessment whenever an eating occasion occurred	Consumption of foods (i.e., dairy, fruit, vegetables, grains, protein, sauce/condiment, soup, and sweets/snacks) and portion size	[58]
Toddler Overweight Prevention Study (TOPS)	277 mother–child pairs	To identify factors in the home environment associated with child diet	Palm device	≤8 days	Signal-contingent assessment at random intervals	Child consumption of meals, snacks or drinks (i.e., desserts, salty foods, fried foods, fruits, vegetables, milk, diet drink, sweetened drink, water)	[59,60]
Wahl 2017	38 adults aged 18 to 48 years	To examine the eating happiness and satisfaction experienced in real-time and in real life	Smartphone	8 days	Event-contingent assessment whenever an eating occasion occurred	Type of meal including a picture of food and a description of its main components	[61]
Wouters 2016	46 students aged 20 to 50 years	To compare a signal-contingent smartphone app with an event-contingent paper and pencil diary for assessing total energy intake	Smartphone	4 days	Signal-contingent assessment at random intervals	Reporting on snacking and drinking (i.e., type and amount) between meals	[62]
			Paper-and-pencil	4 days	Event-contingent assessment whenever an eating occasion occurred	Reporting on snacking and drinking (i.e., type and amount) between meals	
Waki 2014	54 patients with type 2 diabetes randomly allocated into 2 groups	To develop a real-time, partially automated interactive system to interpret patients’ data and respond with appropriate actionable findings, helping the patients achieve diabetes self-management	Smartphone	3 months	Event-contingent assessment whenever an eating occasion occurred	Phone images of all eating occasions	[63]
Zenk 2014	101 women aged 25 to 65 years	To examine contributions of fluctuations in environmental and personal factors to within-person and between-person variations in snack food intake	Web-based survey	7 days	Signal-contingent assessment at random intervals	Consumption of snacks (i.e., cookies or sweetened baked goods, chocolate or candy, ice cream or frozen dessert, salty snacks, and French fries or other fried side dish)	[64]

## Supplementary Materials

Supplementary materials can be found at https://www.mdpi.com/2072-6643/11/11/2696/s1. Table S1: PRISMA 2019 Checklist.
